# Impact of 10-Valent Pneumococcal Conjugate Vaccine on Bacterial Meningitis in Madagascar

**DOI:** 10.1093/cid/ciz504

**Published:** 2019-09-05

**Authors:** Emilson Jean P R Andriatahirintsoa, Julia Liliane Raboba, Vonintsoa Lalaina Rahajamanana, Ando Lalaina Rakotozanany, Mengouom M Nimpa, Yolande Vuo Masembe, Goitom Weldegebriel, Linda De Gouveia, Jason M Mwenda, Annick Lalaina Robinson

**Affiliations:** 1 Teaching Hospital, Centre Hospitalier Universitaire AnosialaAntananarivo, Madagascar; 2 Department of Child Health, Teaching Hospital, Centre Hospitalier Universitaire Mère Enfant TsaralàlanaAntananarivo, Madagascar; 3 World Health Organization (WHO) Country Office, Antananarivo, Madagascar; 4 WHO Intercountry Support Team for East and Southern Africa, Harare, Zimbabwe; 5 Regional Reference Laboratory, Centre for Respiratory Diseases and Meningitis, National Institute of Communicable Diseases, Johannesburg, South Africa; 6 WHO Regional Office for Africa, Brazzaville, Republic of Congo

**Keywords:** acute bacterial meningitis, pneumococcal conjugate vaccine, PCV, *Streptococcus pneumoniae*

## Abstract

**Background:**

The 10-valent pneumococcal conjugate vaccine (PCV10) was introduced in Madagascar in 2012. The objective of this study was to determine the impact of PCV10 on bacterial meningitis in hospitalized children <5 years of age.

**Methods:**

During 2010–2017, data from the hospital admission logbook were recorded for bacterial meningitis and pneumonia hospitalizations in children <5 years of age. Between April 2011 and December 2017, 3312 cerebrospinal fluid (CSF) samples collected from children who fulfilled the World Health Organization case definition of suspected bacterial meningitis were analyzed at the sentinel site laboratory (SSL) by microscopy, culture, and antigen detection tests. A total of 2065 CSF samples were referred to the regional reference laboratory for real-time polymerase chain reaction (RT-PCR) analysis. 2010–2011 was defined as the prevaccine period, 2012 as vaccine introduction year, and 2013–2017 the postvaccine period. The number of cases, causative agent, and pneumonia hospitalizations were compared before and after PCV10 introduction.

**Results:**

In the prevaccine period, bacterial meningitis and pneumonia hospitalizations accounted for 4.5% and 24.5% of all hospitalizations while there were 2.6% and 19%, respectively, in the postvaccine period (*P* < .001). In samples tested at the SSL, 154 were positive with 80% *Streptococcus pneumoniae* and 20% other bacteria. Pneumococcal meningitis diagnosed by RT-PCR declined from 14% in 2012 to 3% in 2017. Also, 14% of children with pneumococcal meningitis died.

**Conclusions:**

Following PCV10 introduction, pneumococcal meningitis, bacterial meningitis, and pneumonia hospitalizations declined. Surveillance should continue to monitor the impact of PCV10.

## Teaser

We found a decline in pneumococcal meningitis, bacterial meningitis, and pneumonia hospitalizations after the introduction of pneumococcal conjugate vaccine (PCV). It is essential to continue conducting such sentinel surveillance to monitor the long-term impact of 10-valent PCV.

Meningitis is a devastating infection associated with high mortality and morbidity [1]. In 2002, there were an estimated 1 730 000 deaths due to meningitis, and the majority were among children in the developing world [2, 3]. In 2008, an estimated 541 000 children <5 years old died from pneumococcal disease worldwide. Africa accounted for 57% of these deaths [4]. *Streptococcus pneumoniae* is a leading cause of bacterial meningitis among children <5 years old, followed by *Haemophilus influenzae* type b (Hib) and *Neisseria meningitidis* [2].

A total of 139 (72%) countries globally had introduced pneumococcal conjugate vaccine (PCV) into their national routine infant immunization schedules by December 2017 [5]. The 10-valent PCV (PCV10) was included into the Extended Programme on Immunization in Madagascar in October 2012 and is recommended as a 3-dose vaccination schedule (3 + 0) at 6, 10, and 14 weeks of life. Madagascar national coverage of PCV10 was 76% in 2013, 72% in 2014, 69% in 2015, 76% in 2016, and 74% in 2017 [6].

Since PCV introduction, limited studies are available showing impact of PCV in Africa. This is the first evaluation of PCV10 performance in Madagascar. We evaluated the impact of PCV10 on bacterial meningitis and pneumonia hospitalizations in Madagascar.

## METHODS

### Study Site

Centre Hospitalier Universitaire Mère Enfant Tsaralalàna (CHUMET) is a referral pediatric hospital with 82 beds. It is a sentinel site for surveillance of pediatric bacterial meningitis in Antananarivo, the capital of Madagascar. CHUMET is the only sentinel surveillance site in Madagascar that reports to the World Health Organization (WHO) sentinel hospital surveillance network. It receives patients both from the capital city and its surroundings as well as from the provinces and districts when referred.

### Study Design and Case Definition

We analyzed all admissions, bacterial meningitis, and pneumonia hospitalizations in children <5 years of age who were admitted to CHUMET. Any child aged 0–59 months admitted to the sentinel hospital with sudden onset of fever (>38.5°C rectal or 38.0°C axillary) and with neck stiffness, altered consciousness with no other alternative diagnosis, or other meningeal sign was eligible for inclusion.

Sentinel surveillance data were collected on pediatric bacterial meningitis from April 2011 to December 2017. The time periods were categorized as follows: pre–PCV10 introduction (1 January 2010 through 31 December 2011); the year of vaccine introduction (2012); and the post–PCV period, which includes the 5 years after vaccine introduction (1 January 2013 through 31 December 2017). When comparing the prevaccine against the postvaccine period, the data from 2012 (year of PCV introduction) were not included in the analysis.

### Data and Sample Collection

Sociodemographic and clinical information was collected using a standard WHO case investigation form. A cerebrospinal fluid (CSF) sample was collected from children who fulfilled the WHO case definition of suspected bacterial meningitis between 2011 and 2017 [7].

### Hospital Logbook Review

We reviewed the hospitalization admission logbooks from 2010 to 2017 and recorded total admissions in children <5 years old as well as the number of children admitted for bacterial meningitis and pneumonia.

### Laboratory Methods

CSF samples were processed at the sentinel site laboratory (SSL) microscopically by Gram stain. Routine diagnostic testing (culture and/or antigen detection test and/or BinaxNOW) was performed on CSF samples, at SSL, for determination of the etiology of bacteria causing the meningitis. Since 2012, any residual CSF samples were referred to the regional reference laboratory (RRL) at the National Institute of Communicable Diseases in South Africa for further analysis by real-time polymerase chain reaction (PCR) to detect pneumococcus, meningococcus, and *H. influenzae*. PCR assays were performed using the Applied Biosystems 7500 Fast real-time PCR platform (Applied Biosystems). For DNA extractions, the MagNA Pure 96 instrument (Roche) was used and a multiplex real-time PCR assay was run for the molecular detection of meningitis pathogens targeting the *ctrA*, *lytA*, and *hpd* genes for *N. meningitidis*, *S. pneumoniae*, and *H. influenzae*, respectively [8].

### Data Analysis

Data were entered into Microsoft Excel and analyzed using R statistical software. We compared the number of bacterial meningitis cases, causative agent, and pneumonia hospitalizations before and after PCV10 introduction and assessed the differences between characteristics of pneumococcal meningitis in vaccinated and unvaccinated children.

Quantitative variables were represented as percentage. To compare the variables, we used the χ ^2^ and Fisher exact tests. A significant association was defined as *P* < .05.

## RESULTS

**Figure 1.**
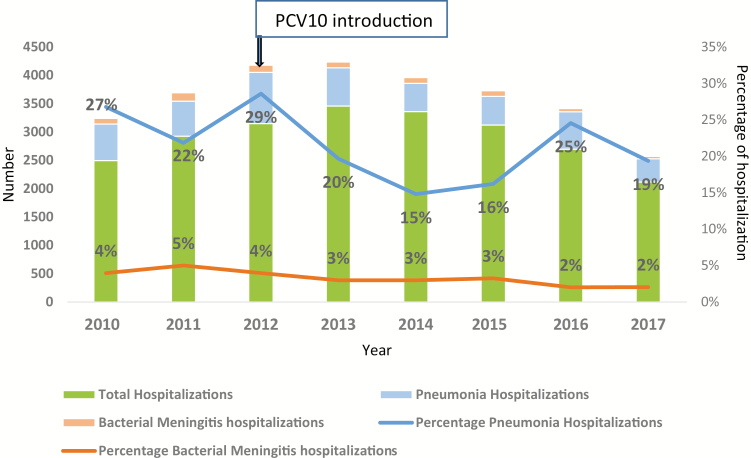
Percentage of total hospitalizations due to meningitis and pneumonia, 2010–2017, Madagascar. Abbreviation: PCV10, 10-valent pneumococcal conjugate vaccine.

From January 2010 through December 2017, a total of 23 327 children <5 years of age were hospitalized in the pediatrics ward at CHUMET. Bacterial meningitis hospitalization contributed to 4.5% on average in the prevaccine period (2010–2011) and an average of 2.6% of hospitalizations after introduction (2013–2017). The percentage reduction in hospitalizations due to bacterial meningitis post–PCV10 introduction was 42% (*P* < .001; Figure 1). During the same period, 4974 children were hospitalized due to pneumonia. The average proportion of pneumonia hospitalization from all hospitalizations was 24.5% in the prevaccine years of 2010–2011 and 19% in 2013–2017 post–PCV10 introduction. The percentage reduction in pneumonia hospitalizations is 22% and this was statically significant (*P* < .001; Figure 1).

From April 2011 through December 2017, we collected 3312 CSF samples from children <5 years old hospitalized at CHUMET. Using 1 or more routine diagnostic tests, different bacteria were identified in 154 CSF samples tested at SSL. Of those, 122 (about 80%) were positive for *S. pneumoniae* and 32 (about 20%) were positive for other bacteria such as Hib, *N. meningitidis*, group B streptococci, and others.

**Figure 2.**
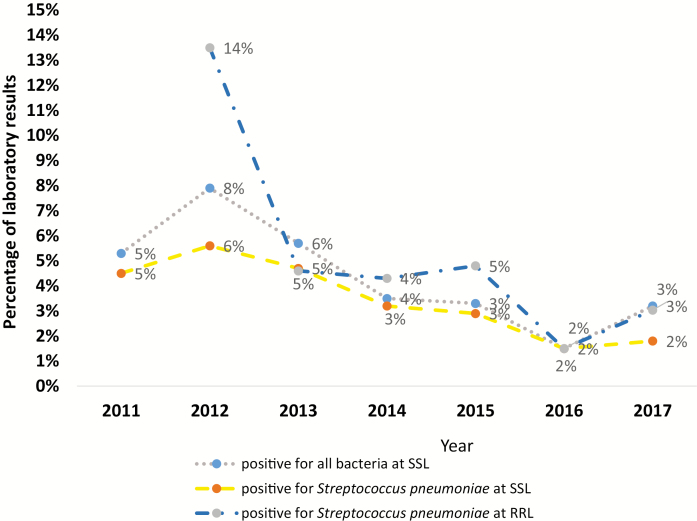
Bacteria identified from cerebrospinal fluid samples tested between 2011 and 2017 at sentinel site laboratory (SSL) Centre Hospitalier Universitaire Mère Enfant Tsaralalàna (CHUMET) and at the regional reference laboratory (RRL) by polymerase chain reaction (source: laboratory data register, CHUMET and RRL South Africa).

Since 2012, a total of 2065 CSF samples were referred to the RRL for further analysis. The percentage of specimens positive for *S. pneumoniae* by real-time PCR declined from 28 of 208 samples tested (14%) in 2012 to 5 of 165 samples tested (3%) in 2017 (Figure 2).

In this study, the vaccine coverage for children <5 years of age that fulfilled WHO definition of suspected bacterial meningitis (vaccination information by history and from vaccination card) and received at least 2 doses of PCV10 was 41% in 2013 and 75%–86% from 2014 through 2017. However, only 14% of them confirmed their vaccination status by vaccination card; the remaining statuses were obtained through verbal reports from caregivers (Table 1).

**Table 1.** Nationwide 10-Valent Pneumococcal Conjugate Vaccine Coverage and Vaccine Coverage in Children With Suspected Bacterial Meningitis in Madagascar, 2013–2017

Coverage	Year				
	2013	2014	2015	2016	2017
Madagascar national EPI coverage, No.	76%	72%	69%	76%	74%
Vaccination status of children with suspected meningitis at CHUMET					
Vaccine information by history and/or from vaccination card, No. (%)	124 (41)	301 (86)	370 (75)	383 (84)	202 (79)
Vaccine information from vaccination card, No. (%)	2 (1.6)	9 (2.9)	75 (203)	75 (19.6)	35 (17.3)

Abbreviations: CHUMET, Centre Hospitalier Universitaire Mère Enfant Tsaralalàna; EPI, Expanded Programme on Immunization.

We evaluated the characteristics of the 122 patients who tested positive for pneumococcal meningitis (Table 2). Children aged between 1 month and <l year were most affected by pneumococcal meningitis (71%), followed by those aged between 1 year and <3 years (18%); 68 (56%) of all patients were male and 54 (44%) female (sex ratio, 1.3; Table 2).

**Table 2.** Clinical and Demographic Characteristics of Pneumococcal Meningitis Cases, 2012–2017

Characteristic	All Cases (N = 122)
	No. (%)
Age group	
Neonate	4 (3)
1 mo to <1 y	86 (70)
1 y to <3 y	22 (18)
3 y to <5 y	10 (8)
Sex	
Male	68 (56)
Female	54 (44)
Clinical signs	
History of fever	113 (93)
Presence of seizure	78 (64)
Altered consciousness	34 (28)
Respiratory sign	43 (35)
Neck stiffness	13 (11)
Bulging fontanel	11 (9)
Vaccination status	
Card confirmed	11 (9)
Verbal report	32 (26)
Not vaccinated	79 (65)
Outcome	
Died	17 (14)

Almost all children (n = 113 [93%]) had history of fever, and more than half (n = 78 [63%]) had seizure. Other common clinical signs included neck stiffness (n = 13 [11%]), altered consciousness (n = 34 [43%]), and bulging fontanel (n = 11 [9%]).

The majority of the children positive for *S. pneumoniae* were not vaccinated with PCV10 (n = 79 [65%]); 11 (9%) cases were vaccinated with PCV10 and confirmed by vaccination card (3 cases received 1 dose and 8 cases received 3 doses) and 32 (26%) were vaccinated with PCV10 as reported by a caregiver. Overall, 17 (14%) of all children affected by pneumococcal meningitis died (Table 2).

## DISCUSSION

This is the first evaluation of the long-term impact of PCV10 on bacterial meningitis and pneumonia hospitalizations 5 years after vaccine introduction in Madagascar. We found a 42% reduction of bacterial meningitis and 22% reduction of pneumonia hospitalizations after introduction of PCV into the routine immunization program. The trend in pneumonia hospitalization paralleled the pattern in hospitalizations due to bacterial meningitis.

During the first 5 years, PCV coverage was between 69% and 76% nationally in Madagascar. The PCV coverage for Africa in 2017 was 68% [6].

Total hospitalization recorded in admission books remained stable in the years 2011–2014 and started to decline from 2015 to 2017. Hospital admissions in 2016 and 2017 were the lowest since 2011. Such a decrease in total hospitalizations can be attributed to introduction of PCV in 2012 and Rotarix vaccine in May 2014. A reduction in total hospitalizations due to diarrhea was noted in Madagascar from 25% (before vaccine introduction) to 16% (after vaccine introduction), which is a 36% reduction in diarrhea hospitalizations post–rotavirus vaccine period [9]. The decrease in detection of *S. pneumoniae* by routine bacteriology test at the sentinel site hospital as well as real-time PCR supplements the decline in pneumonia and bacterial meningitis hospitalizations. We acknowledge that our findings can be influenced by the general progressive improvements in prevention, healthcare, and treatment during the evaluation period. The country has been implementing various child survival interventions during the annual African vaccination week where mass deworming and vitamin A supplementations are implemented and the benefits of breastfeeding, handwashing, and minimizing indoor air pollution are highly promoted (Ministry of Health Madagascar, unpublished data) [10, 11].

Our findings are similar to those reported in other settings where impact of PCV was seen. The incidence of pneumococcal disease including bacterial meningitis quickly declined in high-, middle-, and low-income countries such as the United States, England, Australia, South Africa, and The Gambia, and in Europe [12, 17]. Such reduction was observed both in the vaccinated and unvaccinated age groups, suggesting that PCV use has indirect effects, for example, in South Africa [12].

The main causative agents of bacterial meningitis are Hib, *S. pneumoniae*, and *N. meningitidis* [18, 19]. We detected a variety of bacteria, but *S. pneumoniae* remained the leading causative agent even after introduction of the vaccine. We believe that this is expected since non-PCV serotypes will continue to cause some level of morbidity.

The case fatality rate (CFR) of pneumococcal meningitis is high (10%–30%), mainly affecting children aged <2 years [20, 21]. A mortality ratio of 13.7% was observed in Tunisia [22], which is similar to our findings whereby CFR was 14% in children with CSF samples positive for *S. pneumoniae*.

The strength of our study is that it used multiple data sources such as logbook review of total admissions vs pneumonia and meningitis admissions over an extended period of time. This was complemented by bacteriology and molecular test results of CSF samples. However, our study also has some limitations. First, the study was conducted in children hospitalized for suspected meningitis in a sentinel teaching hospital, and these findings may not be generalizable to the wider Malagasy population as the sentinel site is located in an urban setting. Second, we monitored national vaccination coverage and acknowledge that some areas are less served by immunization; in particular, optimal vaccine coverage may not have been achieved in hard-to-reach and inaccessible areas, and children living in those areas may still be affected by bacterial meningitis and *S. pneumoniae* to a higher degree. Last, only 1 year of data was available before PCV10 introduction as Madagascar joined the sentinel surveillance network in 2011.

## CONCLUSIONS

This is the first study to show evidence of the impact of PCV10 on bacterial meningitis in Madagascar. We found a decline in pneumococcal meningitis, bacterial meningitis, and pneumonia hospitalizations after the introduction of PCV. It is essential to continue conducting such sentinel surveillance to monitor the long-term impact of PCV10.

## Notes

***Acknowledgments.*** The authors thank the Centre Hospitalier Universitaire Mère Enfant Tsaralalàna staff; Expanded Program Immunisation Madagascar; the South African National Institute of Communicable Diseases; and the World Health Organization.

***Financial Support.*** Financial support for sentinel site surveillance was provided by the Madagascar Ministry of Health, Gavi - the Vaccine Alliance, through a grant to the WHO for the African Paediatric Bacterial Meningitis Surveillance Network.

***Supplement sponsorship.*** This supplement was supported with funds from Gavi, the Vaccine Alliance through The World Health Organization and the CDC Foundation, and The Medical Research Council Unit The Gambia at the London School of Hygiene and Tropical Medicine.

***Potential conflicts of interest.*** The authors: No reported conflicts of interest. All authors have submitted the ICMJE Form for Disclosure of Potential Conflicts of Interest. Conflicts that the editors consider relevant to the content of the manuscript have been disclosed.
